# Biochemical Correction of GM2 Ganglioside Accumulation in AB-Variant GM2 Gangliosidosis

**DOI:** 10.3390/ijms24119217

**Published:** 2023-05-24

**Authors:** Natalie M. Deschenes, Camilyn Cheng, Alex E. Ryckman, Brianna M. Quinville, Prem Khanal, Melissa Mitchell, Zhilin Chen, Waheed Sangrar, Steven J. Gray, Jagdeep S. Walia

**Affiliations:** 1 Centre for Neuroscience Studies, Queen’s University, Kingston, ON K7L 3N6, Canada; camilyn.cheng@queensu.ca (C.C.); alex.livie.ryckman@gmail.com (A.E.R.); 13bq@queensu.ca (B.M.Q.);; 2 Department of Pediatrics, Queen’s University, Kingston, ON K7L 2V7, Canada; pk46@queensu.ca (P.K.); mdm10@queensu.ca (M.M.); 3 Department of Biomedical and Molecular Sciences, Queen’s University, Kingston, ON K7L 3N6, Canada; zc@queensu.ca; 4 Department of Pediatrics, UT Southwestern Medical Center, Dallas, TX 75390, USA; steven.gray@utsouthwestern.edu

**Keywords:** GM2 gangliosidosis, gene therapy, adeno-associated viral vectors, GM2 ganglioside, neurodegeneration, dose-response, intrathecal, AAV9, GM2 activator protein

## Abstract

GM2 gangliosidosis is a group of genetic disorders that result in the accumulation of GM2 ganglioside (GM2) in brain cells, leading to progressive central nervous system (CNS) atrophy and premature death in patients. AB-variant GM2 gangliosidosis (ABGM2) arises from loss-of-function mutations in the GM2 activator protein (GM2AP), which is essential for the breakdown of GM2 in a key catabolic pathway required for CNS lipid homeostasis. In this study, we show that intrathecal delivery of self-complementary adeno-associated virus serotype-9 (scAAV9) harbouring a functional human *GM2A* transgene (*scAAV9.hGM2A*) can prevent GM2 accumulation in in GM2AP-deficient mice (*Gm2a*^−/−^ mice). Additionally, *scAAV9.hGM2A* efficiently distributes to all tested regions of the CNS within 14 weeks post-injection and remains detectable for the lifespan of these animals (up to 104 weeks). Remarkably, GM2AP expression from the transgene scales with increasing doses of *scAAV9.hGM2A* (0.5, 1.0 and 2.0 × 10^11^ vector genomes (vg) per mouse), and this correlates with dose-dependent correction of GM2 accumulation in the brain. No severe adverse events were observed, and comorbidities in treated mice were comparable to those in disease-free cohorts. Lastly, all doses yielded corrective outcomes. These data indicate that *scAAV9.hGM2A* treatment is relatively non-toxic and tolerable, and biochemically corrects GM2 accumulation in the CNS—the main cause of morbidity and mortality in patients with ABGM2. Importantly, these results constitute proof-of-principle for treating ABGM2 with *scAAV9.hGM2A* by means of a single intrathecal administration and establish a foundation for future preclinical research.

## 1. Introduction

GM2 gangliosidosis is a group of fatal autosomal, recessive lysosomal-storage disorders (LSDs) in which GM2 gangliosides (GM2)—a type of glycosphingolipid found primarily in the central nervous system (CNS)—accumulate to cytotoxic levels in the lysosomes of neurons. Chronic GM2 storage in lysosomes drive CNS-wide apoptosis, which leads to progressive CNS dysfunction, and ultimately, CNS failure and death. Hence, intralysosomal GM2 accumulation is the principal biochemical hallmark of morbidity and lethality for this group of LSDs.

The hydrolase β-N-acetylhexosaminidase A (β-HexA) is one of many lysosomal glycohydrolases that breakdown gangliosides in conserved endocytosis-regulated catabolic pathways that regulate lipid homeostasis in the CNS. β-HexA is a heterodimeric polypeptide composed of α- and β-subunits encoded by two distinct genes (*HEXA* and *HEXB*, respectively) [1]. Though there are two other isozymes—HexB and HexS—β-HexA is the only one that specifically hydrolyses GM2 in humans; and this activity requires the stimulatory activity of GM2-activator protein (GM2AP), an essential substrate-specific co-factor that potentiates β-HexA hydrolytic activity against GM2 [2]. GM2AP specifically binds and removes GM2 from lysosomal membranes and presents GM2 to β-HexA for its hydrolysis [3,4]. Structure-function studies show that key features exclusive to the catalytic site in the α-subunit of β-HexA allow it to specifically interact with GM2 and GM2AP within this soluble substrate complex. These features include a positively-charged binding pocket that mediates binding to unique negatively-charged sialic-acid residues in GM2, and a critical loop structure required to interact with GM2AP [4,5,6,7,8]. These structural properties give the β-HexA α-subunit catalytic site hydrolytic specificity against the terminal beta-linked N-Acetylgalactosamine (GalNAc) residue of GM2. Thus, cleavage of GM2 is a key intermediate catabolic step specific to β-HexA and requires synthesis and assembly of three gene products—the α- and β-subunits of β-HexA and GM2AP. This study focused on primary GM2 accumulation manifesting as a diseased state and not on comparatively milder secondary GM2 accumulation reported in many other lysosomal storage disorders, such as Niemann–Pick disease types A, B and C and in in mucopolysaccharidoses such as Hurler, Hunter, Sanfilippo and Sly syndromes [9,10,11,12,13].

Destabilizing mutation(s) in *HEXA* and *HEXB* that reduce expression or activity of their respective α- and β-subunit gene products are associated with aberrant GM2 hydrolysis by β-HexA [14,15], which gives rise to intralysosomal GM2 accumulation in patients with GM2 gangliosidosis. Tay–Sachs disease (TSD) and Sandhoff disease (SD) are two types of clinically identified GM2 gangliosidosis disorders caused by mutations in *HEXA* and *HEXB*, respectively. AB-variant GM2 gangliosidosis (ABGM2), the third type of GM2 gangliosidosis that was discovered by Konrad Sandhoff [16], is caused by mutation(s) in *GM2A*. Of the three disorders, ABGM2 is the rarest and likely the most underdiagnosed type, with less than 30 reported cases [17,18,19,20]. Studies show that patients diagnosed with ABGM2 have normal β-HexA enzyme levels, but harbour homozygous mutations that reduce GM2AP stability and expression, resulting in impaired GM2 breakdown efficiency by β-HexA. Thus, similarly to TSD and SD, ABGM2 is also associated with GM2 storage in the lysosomes of patients. Concordantly, all three GM2 gangliosidosis disorders result in gliosis and neuronal loss, leading to neurological degeneration, developmental arrest, and regression of previously attained motor skills [20,21].

Three forms of TSD, SD, and ABGM2 have been delineated based on their time of onset and severity in the clinic. These forms include the rare adult and juvenile forms, and the more common (acute) infantile form [21]. Disease onset and severity of these forms depend on effective β-HexA and GM2AP function. In the case of ABGM2, effective β-HexA function is compromised to varying extents depending on the underlying loss-of-function mutation in GM2AP. The infantile forms of all three forms have the lowest level of effective β-HexA or GM2AP activity (<2%); correspondingly, these patients are diagnosed the earliest—within 6–14 months of birth—and experience greater disease severity, with death occurring by four years of age [20,21]. In contrast, patients with the juvenile and adult forms have comparatively higher effective β-HexA activities (5–15%) and correspondingly less severe disease, with time of onset to death ranging from 2–10 years for the juvenile form and 21–60 (plus) years for the adult form [22,23,24]. Regardless of the form in which these disorders present, patients experience substantial decline in quality of life, particularly infants; inevitably, all patients will face premature death as there is currently no curative treatment available. While progress has been made in the development of gene therapies for TSD and SD (NCT04798235 and NCT04669535), the development of gene therapies for ABGM2 lag behind, and currently there are no effective treatments available for ABGM2.

ABGM2 is a monogenic disease, making it a feasible candidate for gene replacement therapy. Recent advancements in viral technology have accelerated clinical progress in the development of gene therapies for neurological disorders [25]. Of the viral platforms suitable for gene therapy, adeno-associated viral vectors (AAVs) have emerged as the state-of-the-art method for delivering transgenes to the CNS. AAVs have the ability to transduce dividing and non-dividing cells, and have demonstrated stable transgene expression in the human brain for more than 10 years [26]. Additionally, AAVs have a relatively low pro-inflammatory profile [27], and can express transgene episomally, thereby decreasing the probability of oncogenic events because of low genome integration rates [28,29]. Of the naturally occurring AAV variants, serotype 9 (AAV9) is the most promising for neurologically-based gene therapies because it preferentially targets the brain and spinal cord [30]; unlike other serotypes, it is ‘axonal transport competent’ [31]. Thus, AAV9 enables widespread transgene delivery efficiency throughout all regions of the CNS [31,32,33].

**Figure 1:**
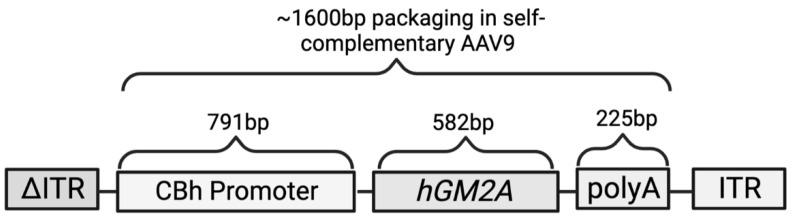
*scAAV9.GM2A* construct design: Inverted terminal repeats (ITRs) flank the promoter, transgene and polyadenylation sequence (polyA). One of the ITRs is mutated (Δ) to create scAAV. The promoter is composed of the cytomegalovirus early enhancer element and a shortened chicken β-actin promoter, termed the CBh [37]. hGM2A, human *GM2A* activator gene; polyA, polyadenelation sequences; Bp, base pairs.

In this report, we assess the potential of a self-complementary (sc) AAV9 vector harbouring a functional human *GM2A* transgene (*scAAV9.hGM2A*; Figure 1) to biochemically rescue β-HexA-dependent GM2 breakdown in a GM2AP-deficient model of ABGM2 (*Gm2a*^−/−^ mice). These mice have normal lifespans and mild ABGM2-like pathologies consistent with weak-to-moderate GM2 accumulation in their brain by 20 weeks of age [34]. Here, *scAAV9.hGM2A* was intrathecally administered in *Gm2a*^−/−^ mice to minimize immediate immunogenic responses known to impede gene therapy efficacy [35,36]. A range of doses were tested to assess toxicity and to estimate the most effective dose required to achieve maximal reversal of GM2 accumulation in vivo. Lastly, the stability of scAAV9.hGM2A gene therapy was assessed by comparing early treatment responses with responses near the end of life (14 weeks post-treatment versus 96 weeks post-treatment or humane endpoint, respectively). These data suggest that scAAV9.hGM2A is safe and tolerable, and biochemically prevents GM2 accumulation in vivo. These data also provide proof-of-concept for the use of *scAAV9.hGM2A* for treating ABGM2 and pave the way for future studies aimed at enabling a phase 1/2 ABGM2 gene therapy trial.

## 2. Results

### 2.1. scAAV9.hGM2A Efficiently Distributes to the CNS of Gm2a^−/−^ Mice Fourteen Weeks after Treatment and Remains Detectable for Their Lifespan

**Figure 2:**
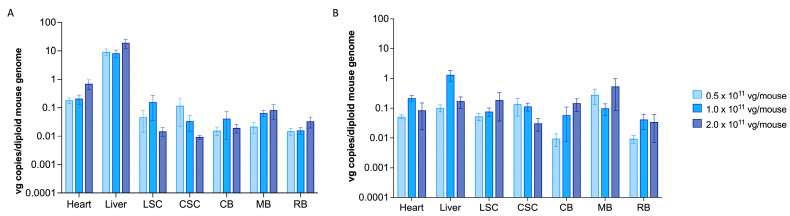
*scAAV9.hGM2A* efficiently biodistributes to CNS, heart and liver and persists for the lifetime of the animals. Adult mice (6 weeks of age) were intrathecally injected with the indicated doses of *scAAV9.hGM2A* (*n* = 6/cohort). LSC, lumbar-section of the spinal cord; CSC, cervical-section of the spinal cord; CB, caudal section of the brain; MB, mid-section of the brain. RB, rostral section of the brain; vg, viral genomes. Data is presented as the number of viral genomes (vector DNA copies) per diploid mouse genome (vg copies/diploid mouse genome). *LaminB2* is used as the internal control. Data are expressed as mean ± SEM. There was no statistically significant difference for DNA copy numbers between *scAAV9.hGM2A* doses in the heart, liver, and the various regions of the CNS. (**A**) *scAAV9.hGM2A* copy number in short-term cohorts—*hGM2A* transgene is detected in the liver, heart, and throughout regions of the CNS 14 weeks post-injection (20 weeks of age); (**B**) *scAAV9.hGM2A* copy numbers in long-term cohorts—*hGM2A* transgene is detectable in the liver, heart, and throughout regions of the CNS 96 weeks post-injection (up to 104 weeks of age, or humane endpoint).

To assess the *scAAV9.hGM2A* biodistribution, a vector (DNA) copy number was calculated based on *GM2A* copies per mouse genome (viral genomes per mouse genome [vg/mouse]). The liver and heart are major AAV-target organs and hence serve as a transduction baseline for inferring the relative copy number for transduction of targeted organs. Consistent with this, *scAAV9.hGM2A* robustly transduced heart and liver for 14 weeks post-injection (1.1–36.8 vg range; 20 weeks of age) (Figure 2A). *scAAV9.hGM2A* viral vector also robustly transduced cells in all regions of the CNS with similar efficacy; however, CNS vector copies were 10- and 100-fold less than heart and liver, respectively (Figure 2A). While trends of dose-dependent infection were apparent in some regions of the CNS, these were not significant, suggesting that all three doses produce comparable levels of vector copies (0.01–0.5 vg per mouse) (Figure 2A). After 96 weeks of treatment (up to 104 weeks of age) vector copies in the liver and heart diminished by over an order of magnitude (~0.02–2.9 vg per mouse genome), consistent with the decreasing titers expected from dilutive effects of ongoing hepatic and cardiac turnover (Figure 2B). However, *scAAV9.hGM2A* copy number in the CNS continued to persist after long-term treatment (~0.01–0.6 vg [long-term treatment] vs. ~0.01–0.5 vg [short-term treatment]) (Figure 2B). Indeed, at 96 weeks post-injection, CNS copies were on par with the diminished copy numbers observed in the liver and heart (Figure 2B). Persistence of the *scAAV9.hGM2A* copy number as late as 104 weeks of age in the CNS reflects long-term subsistence of AAV9 in non-dividing microglial and neuronal cells [38,39]. Collectively, these data suggest that the three tested *scAAV9.hGM2A* doses infect all regions of the CNS at approximately equivalent potency and that this infection persists over the lifespan of these animals.

### 2.2. scAAV9.hGM2A Mediates Expression of Human GM2AP in Gm2a^−/−^ Mice

**Figure 3:**
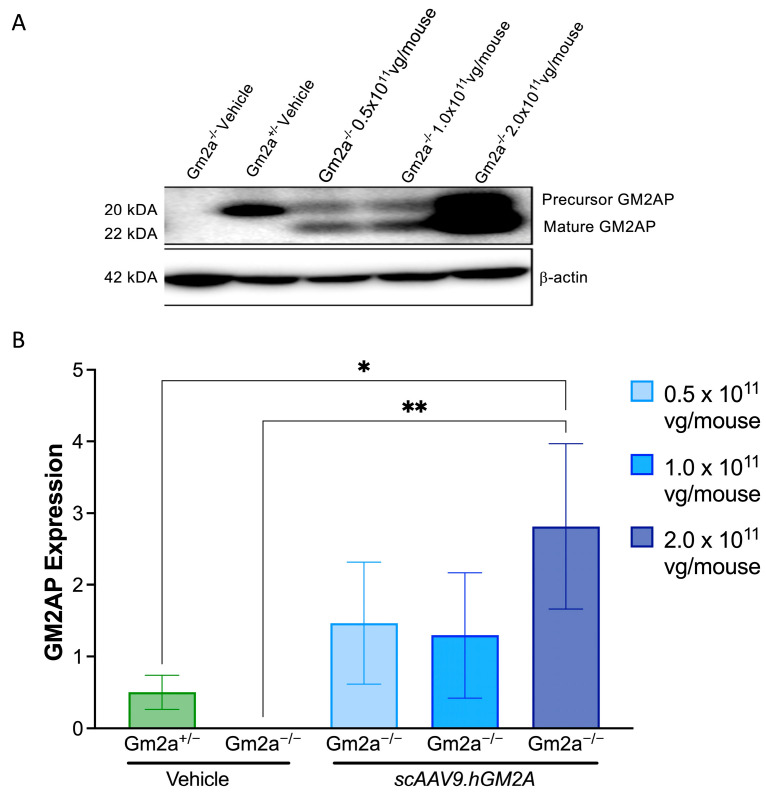
*scAAV9.hGM2A* drives expression of GM2AP in *Gm2a*^−/−^ mice. (**A**) Representative western blot analysis of GM2AP expression in the brains of *Gm2a*^−/−^ mice: Brain mid-sections were dissected from mice 14 weeks after intrathecal administration of the indicated doses of *scAAV9.hGM2A*. The bands migrating at ~20 kDa depict the precursor protein, and the bands migrating at ~22 kDa represent the mature protein. β-actin (42 kDa) was used as an internal control. (**B**) Quantification of the GM2AP signal from the western blots: The sum of intensities of the mature and precursor forms of GM2AP (~20 kDa and 22 kDA, respectively) were taken to represent total GM2AP signal. Intensities were quantified by densitometry and normalized to β-actin intensity (*n* = 3/cohort). The expression level of GM2AP from the highest dose of *scAAV9.hGM2A* was significantly higher than vehicle-treated *Gm2a*^−/−^ mice (2.0 × 10^11^, *p* < 0.0074 [**]; *n* = 3/cohort). The expression level of GM2AP from the 2.0 × 10^11^ vg of *scAAV9.hGM2A* was almost 6-fold greater than the cohort of *Gm2a*^+/−^ disease-free mice, which only showed expression of the endogenous precursor form of GM2AP (22 kDa) (*p* < 0.0252 [*]; *n* = 3/cohort).

To confirm expression of the *GM2A* transgene in the CNS, mid-sections of brain tissue were dissected from *Gm2a*^−/−^ mice 14 weeks post-injection of 0.5, 1.0 and 2 × 10^11^ vg/mouse of *scAAV9.hGM2A* and then analysed by western blotting (Figure 3A,B). As expected, human GM2AP was not detected in vehicle-treated *Gm2a*^−/−^ mice. However, increased levels of human GM2AP protein were detected in brain tissue of the *Gm2a*^−/−^ treated cohort. These levels ranged from 3- to 6-fold higher than endogenously expressed total GM2AP levels in *Gm2a*^−/−^ disease-free mice. Importantly, dose-dependent expression of GM2AP was most apparent after a threshold dose of 1.0 × 10^11^ vg/mouse (Figure 3). These data confirm a correlation between *scAAV9.hGM2A* dose and GM2AP expression in *Gm2a*^−/−^ mice and suggest that *scAAV9.hGM2A* efficiently infects the CNS and mediates GM2AP expression in that organ (Figure 3).

### 2.3. scAAV9.hGM2A Dose-Dependently Diminishes GM2 Accumulation in Gm2a^−/−^ Mice

**Figure 4:**
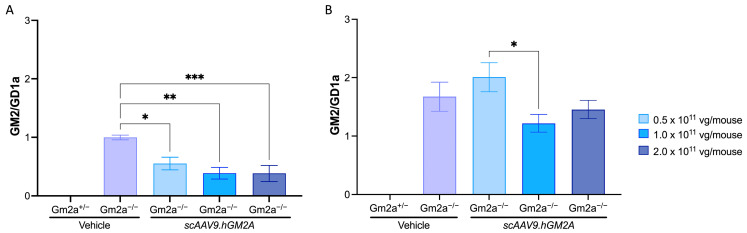
*scAAV9.hGM2A* reduces GM2 ganglioside accumulation in the mid-sections of brains of *Gm2a*^−/−^ mice. *scAAV9.hGM2A* dose-dependently reduces GM2 accumulation 14 weeks post-injection (20 weeks of age), but the potency of this biochemical effect diminishes towards the end of life (96 weeks post-injection; up to 104 weeks of age). GM2 levels are expressed as a function of GD1a, an internal control, which is a ubiquitous ganglioside highly expressed in brain tissue. (**A**) *scAAV9.hGM2A* treatments of 0.5, 1.0 and 2.0 × 10^11^ vg/mouse dose-dependently reduce GM2 ganglioside 14 weeks post-injection (compare with vehicle-treated *Gm2a*^−/−^ cohort; *p* < 0.144 [*], *p* < 0.001 [**] and *p* < 0.0006 [***], respectively; 1-way ANOVA; *n* = 6/cohort). GM2 accumulation is not detected in the disease-free *Gm2a*^+/−^ cohort (*n* = 6). (**B**) Decreases in GM2 ganglioside storage are observed in animals treated 1.0 and 2.0 × 10^11^ vg of *scAAV9.hGM2A* 96 weeks post-injection, however these reductions do not reach significance when compared to untreated *Gm2a*^−/−^ cohort (1-way ANOVA; *n* = 6/cohort). GM2 accumulation is not detected in the disease-free *Gm2a*^+/−^ cohort (*n* = 6).

While biodistribution of a transgene is effective in assessing the transduction efficacy of scAAV9, and detecting GM2AP in CNS is essential to confirm its expression in the tissue of interest, neither is a direct measure of treatment impact, which requires experimental determination of reductions in GM2 accumulation [40]. We, therefore, evaluated the biochemical consequence of *scAAV9.hGM2A*-mediated over-expression of human GM2AP by assessing GM2 storage in the CNS. GM2AP transgene expression correlates with dose-dependent reduction in GM2 accumulation in the mid-section of brains from *Gm2a*^−/−^ mice at 14 weeks post-injection. An average peak reduction of GM2 storage of 2.2-fold was observed with *scAAV9.hGM2A* treatment (Figure 4A; compare cohorts injected with *scAAV9.hGM2A* with vehicle treated *Gm2a*^−/−^ controls). The lowest dose of 0.5 × 10^11^ vg/mouse of *scAAV9.hGM2A* reduced GM2 accumulation by almost 2-fold, while the two higher doses of 1.0 × 10^11^ and 2.0 × 10^11^ vg/mouse of *scAAV9.hGM2A* reduced GM2 accumulation by approximately 2.6-fold. This suggests that 1.0 × 10^11^ vg/mouse of *scAAV9.hGM2A* constitutes likely the most effective dose that can be achieved using this approach in attempt to prevent GM2 accumulation in these mice. As the animals approached the end of life (96 weeks post-injection), GM2 accumulation increased; however, *scAAV9.hGM2A*-treated mice (1.0 × 10^11^ and 2.0 × 10^11^ vg/mouse only) still had almost 0.8-fold less GM2 than vehicle treated *Gm2a*^−/−^ controls (Figure 4B). Notwithstanding, these data clearly show that *scAAV9.hGM2A* can mediate reduction of GM2 accumulation by 14 weeks-post-injection, and moreover, this biochemical correction has potential to persist into the lifetime of the animal, albeit with diminishing potency. Taken together, these data provide proof-of-principle that our *scAAV9.hGM2A* can stably deliver *GM2A* to brain and biochemically reduce GM2 accumulation in a mouse model of ABGM2.

To assess whether correction of GM2 accumulation by GM2AP may therapeutically correct motor behaviour, RR and OFT tests were conducted on vehicle and *scAAV9.hGM2A*-treated cohorts. Except for a single behavioural defect, which showed that *Gm2a*^−/−^ mice require more resting time, a defect that could not be corrected with *scAAV9.hGM2A* (Supplementary Figure S3B), no differences in motor function were detected between vehicle-treated *Gm2a*^−/−^ and *Gm2a*^+/−^ disease-free cohorts over the course of all treatment periods, demonstrating the uninformative nature of these tests in quantifying disease progression in this model (Supplementary Figures S1–S4). These findings suggest that the observed levels of GM2 accumulation in *Gm2a*^−/−^ mice, regardless of treatment, are insufficient to elicit pathological alterations in motor behaviour. This highlights the need to develop a more refined phenotypic mouse model for ABGM2.

**Figure 5:**
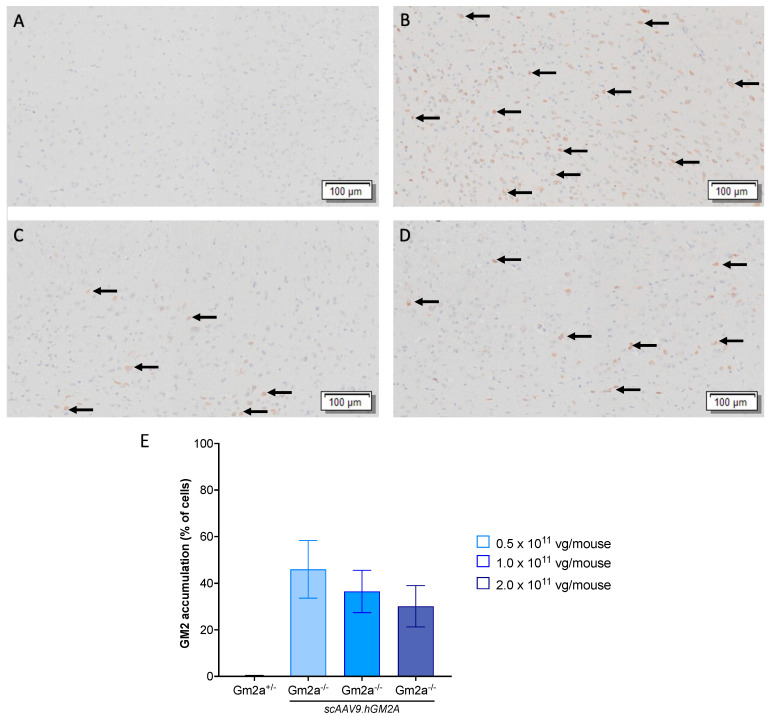
*scAAV9.hGM2A* dose-dependently reduces GM2 accumulation 96 weeks post-injection (up to 104 weeks of age; *n* = 3/cohort). These slides are from central grey matter in the mid-section of the brain and were stained with an anti-GM2 antibody; the brown spots are indicative of GM2 accumulation (noted by the black arrows). The disease-free *Gm2a*^+/−^ cohort (**A**) has no GM2 accumulation, whereas the *scAAV9.hGM2A*-treated *Gm2a*^−/−^ cohorts (**B**–**D**) have mild to moderate accumulation ((**B**): 0.5 × 10^11^ vg; (**C**): 1.0 × 10^11^ vg; (**D**): 2.0 × 10^11^ vg). The cohorts that received 1.0–2.0 × 10^11^ vg of *scAAV9.hGM2A* have visibly less GM2 accumulation than the cohort that received 0.5 × 10^11^ vg of *scAAV9.hGM2A.* (**E**) *scAAV9.hGM2A* treatments of 0.5, 1.0 and 2.0 × 10^11^ vg/mouse dose-dependently reduced GM2 ganglioside 96-weeks post injection. The cohort that received 0.5 × 10^11^ vg of *scAAV9.hGM2A* had significantly more GM2 accumulation than the disease-free *Gm2a*^+/−^ cohort (*p* < 0.0268; *n* = 3/cohort; 1-way ANOVA).

Histological analysis of the mid-section of murine brains demonstrates the dose-responsive reduction in GM2 96-weeks post-injection. There was no visible GM2 accumulation in the short-term cohorts (14 weeks post-injection), as the build-up is relatively moderate in ABGM2 mice [35] (Figures not shown). As shown in Figure 5, there is observable GM2 storage in all treated *Gm2a^−^^/^^−^* mice 96-weeks post-injection, although, GM2 accumulation appeared to be reduced in cohorts that received 1.0 and 2.0 × 10^11^ vg of *scAAV9.GM2A* compared to the cohorts that received 0.5 × 10^11^ vg. These results are consistent with GM2 storage analysis.

### 2.4. scAAV9.hGM2A Is Tolerable over the Lifespan of Treated Animals

**Figure 6:**
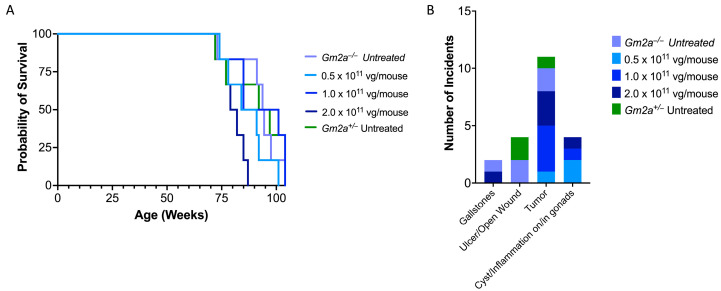
(**A**) Long-term survival of *Gm2a*^−/−^ cohorts treated with *scAAV9.hGM2A* is comparable to the vehicle-treated cohort. Kaplan–Meier survival curve indicates the cohorts of *Gm2a*^−/−^ mice treated for 96 weeks with *scAAV9.hGM2A* did not exhibit significant differences in survival compared to their vehicle-treated counterparts, or to disease-free (*Gm2a*^+/−^) heterozygote controls (*Gm2a*^+/−^ [disease-free], 91 ± 13.7 weeks; *Gm2a*^−/−^ [vehicle], 92.3 ± 10.4 weeks; *Gm2a*^−/−^, 86.7 ± 10.0 [0.5 × 10^11^ vg/mouse]; *Gm2a*^−/−^, 92.2 ± 12.6; [1.0 × 10^11^ vg/mouse]; *Gm2a*^−/−^, 80.8 ± 4.9 weeks [2.0 × 10^11^ vg/mouse]; *Gm2a*^−/−^, 86.7 ± 10.3; [0.5–2.0 × 10^11^ vg; all doses]; Age: [avg. ± SD]; 2-way ANOVA). (**B**) Comorbidities observed in *scAAV9.hGM2A*- and vehicle-treated cohorts of *Gm2a*^−/−^ mice: The number of incidents of various pathologies observed at end-of-life (104 weeks of age) or defined humane endpoints (see methods) are shown. Overall comorbidity incidence in *Gm2a*^−/−^-treated cohorts were similar in number to corresponding comorbidities detected in the vehicle-treated *Gm2a*^−/−^ and *Gm2a*^+/−^ disease-free cohorts.

*Gm2a*^−/−^ mice have normal lifespans [34]; hence, *Gm2a*^−/−^ mice are an ideal model to assess chronic toxicity of *scAAV9.hGM2A*. Consistent with a previous report [34], *Gm2a*^−/−^ mice had an average life expectancy comparable to disease-free controls of the same strain (92 ± 10 vs. 91 ± 14 weeks, respectively) (Figure 6A; compare vehicle-treated *Gm2a*^−/−^ mice to *Gm2a*^+/−^ mice). Treatment of *Gm2a*^−/−^ mice with a range of *scAAV9.hGM2A* doses corresponding to 0.5, 1.0 or 2.0 × 10^11^ vg/mouse did not impact the average life expectancy of *Gm2a*^−/−^ mice relative to vehicle-treated counterparts (Figure 6A; 92 ± 10 weeks of age [vehicle] vs. 87 ± 10. [0.5 × 10^11^ vg], 92 ± 13 [1.0 × 10^11^ vg] and 81 ± 5 [2.0 × 10^11^ vg] weeks of age). Indeed, the average lifespan of all *scAAV9.hGM2A*-treated animals calculated across the range of doses was 87 ± 10 weeks of age. These data show that survival is not significantly impacted by treatment across a wide range of *scAAV9.hGM2A* doses, suggesting that biochemical prevention of GM2 accumulation by this gene therapy is safe and tolerable as a one-time gene therapy treatment over the lifetime of these animals.

### 2.5. Similar Comorbidity Profiles of scAAV9.hGM2A-Treated and Untreated Cohorts Support Its Safety as a Gene Therapy

Incidents of comorbidity were tracked over the course of *scAAV9.hGM2A* treatment (Figure 6B). The number of incidents of morbidity in *Gm2a*^−/−^ mice over 96 weeks of treatment were minor, as suggested by similar morbidity incidence in vehicle-treated *Gm2a*^−/−^ and *Gm2a*^−/−^ disease-free controls. Of the comorbidities tracked, threshold weight loss (≥15%) constituted the major comorbidity, however, the number of these incidents in *Gm2a*^−/−^ treated cohorts (2–4 incidents) are near-identical to those observed in the vehicle-treated *Gm2a*^−/−^ and disease-free cohorts (3 incidents, respectively). The second major comorbidity was tumorigenesis (Figure 6B); tumours were detected in 33% of mice, in which spleen (44.4%) and liver (22.2%) were the predominant sites of onset. Tumours’ incidence in *Gm2a*^−/−^–treated cohorts did not track with dose (1–4 incidents); and importantly, incident numbers are comparable to vehicle-treated *Gm2a*^−/−^ and disease-free cohorts (2 and 1 incident(s), respectively) (Figure 6B). The remaining comorbidity types tracked in *Gm2a*^−/−^-treated cohorts were overall less prominent and—similar to the weight-loss and tumour onset incidents—approximated in type and number the comorbidities arising in vehicle-treated *Gm2a*^−/−^ and *Gm2a*^−/−^ mice (Figure 6B). These data show that the comorbidity profiles of treated cohorts are comparable to those of vehicle-treated controls and suggests that chronic *scAAV9.hGM2A* treatment at the tested doses of 0.5, 1.0 or 2.0 × 10^11^ vg/mouse are likely not associated with significant toxicity.

## 3. Discussion

In the present study regarding the intrathecal route of administration and gene therapy dose-response, we assessed the efficacy of *scAAV9.hGM2A* as a potential gene therapy for ABGM2. A single injection of *scAAV9.hGM2A* at 6 weeks of age (murine early adult stage) resulted in a discernible expression of GM2AP in the brain, and in turn, reduced accumulation of GM2. Vector copies were detectable in all examined regions of the CNS at 14 weeks post-treatment. It is worth noting that these vector copies were significantly lower, approximately 10–100 fold less, than those observed in the heart and liver, which are well-known targets of systemic AAV infection [41,42,43,44] (Figure 2A). Generally, *scAAV9.hGM2A* vector copies in the CNS remained relatively consistent 96 weeks post-treatment; however, heart and liver copy numbers diminished over time, likely because of cell turnover in these organs (Figure 2B). The long-lived stability of *scAAV9.hGM2A* in the CNS is likely attributable to the non-dividing neuronal cells of the CNS, the lifespans of which can last the subject’s lifetime. Moreover, copy numbers in various regions of the CNS were similar, regardless of the dose administered, suggesting that these doses are within the ranges necessary to achieve maximal CNS infection, or alternatively that the differences between them are not discernible with the numbers of animals used in this study. Thus, one-time intrathecal administration of *scAAV9.hGM2A* at doses ranging from 0.5–2.0 × 10^11^ vg/mouse appears to stably transduce target cells across the CNS, and the transgene expression persisted over the lifespan of the animals, though the effect appeared to plateau after the middle dose (1.0 × 10^11^ vg/mouse). An important caveat to note is that, even at 2.0 × 10^11^ vg/mouse, CNS biodistribution values were below 1 vg copy per mouse genome. Thus, the maximum dose remains sub-saturating in terms of overall CNS gene transfer, which conceptually would argue that the highest safe dose would be advisable in order to achieve the maximum therapeutic benefit.

The *scAAV9.hGM2A* biodistribution data reported here are consistent with prior studies on intrathecally administered AAV-mediated therapy [45,46]. These studies suggest that stable transgene expression can be detected up to a year post-injection at a dose of 0.5 × 10^11^ vg/mouse [46]. Notably, the dose range of 0.5–2.0 × 10^11^ vg/mouse tested in the present study is well below ranges explored in other investigations reporting biochemical correction of aberrant genes in neurodegenerative disorders (0.4–5.0 × 10^12^ vg) [30,47,48]. Moreover, the dose range used in this study produces viral copy numbers that surpass those achieved with intravenous injection of AAV9-based therapies for SD and TSD, which are plagued by toxicity and therapy-interfering immune responses because higher doses are necessary to achieve comparable infection efficacy in the CNS [43,49]. Hence, these data support intrathecal administration as a safer and more efficacious CNS transduction route than intravenous delivery. Indeed, gene therapy trials for neurological disorders have recently been opened that employ intrathecal administration (e.g., NCT05606614, NCT05394063, NCT05089656), including the first in-human phase 1/2 trial for TSD and SD (NCT04798235).

Expression of GM2AP was detected in the mid-section of brains with all three doses of *scAAV9.hGM2A* 14 weeks after injection (Figure 3). The lower two doses (0.5–1.0 × 10^11^ vg) produced similar levels of GM2AP expression, and the highest dose (2.0 × 10^11^ vg) increased GM2AP expression levels by two-fold. While these expression data are not—strictly speaking—exemplary of dose-dependent behaviour, they are suggestive of such an effect. Idealized dose-dependency in this case could be masked by several methodological challenges; these include limitations associated with administering fixed doses independent of weight, which at the time of injection, ranged from 15–23 g. Notwithstanding, the dose-dependent-like GM2AP expression pattern observed in these experiments correlate with dose-dependent biochemical correction of GM2 accumulation in *Gm2a*^−/−^ mice after 14 weeks (Figure 4). This demonstrates that intralysosomal GM2 accumulation in the CNS arising from GM2AP-deficiency can be dose-dependently corrected in vivo by replacement with a human *GM2A* transgene encoding GM2AP. These data also suggest that 1.0 × 10^11^ vg/mouse of *scAAV9.hGM2A* is likely the most effective dose that can be achieved with this vector, as it is equally as effective at inhibiting GM2 accumulation as is the higher dose used in this study (2.0 × 10^11^ vg/mouse). We did not notice any toxicity related to overexpression in the highest dose (2.0 × 10^11^ vg/mouse) in terms of neuronal death or regression in motor function.

One might expect a reduction in GM2 accumulation with *scAAV9.hGM2A* in *Gm2a*^−/−^ mice to correlate with improved motor function, further predicting a phenotypic improvement in patients with ABGM2. However, we were unable to conclude this because we did not observe significant differences in motor behaviour between vehicle-treated *Gm2a*^−/−^ and *Gm2a*^+/−^ disease-free controls (Supplementary Figures S1–S4). This suggests that GM2 accumulations in *Gm2a*^−/−^ mice, although detectable and correctable, are insufficiently elevated to induce overt motor pathologies characteristic of the human form of ABGM2, or of other murine models of GM2 gangliosidosis [50]. Thus, the observed rescue of the GM2 storage defect in these studies may represent a correction of comparatively lower levels of intralysosomal GM2: levels that remain within the limits of detection of the ganglioside assay but are non- or mildly-pathological. This is consistent with the initial characterization of *Gm2a*^−/−^ mice in which weak motor behaviour impairment was attributed to low quantities of GM2 accumulation relative to amounts that accumulate in murine models of SD [34]. Interestingly, low levels of GM2 accumulation in this model have been attributed to an alternate pathway that is less prominent in humans, which enables GM2 ganglioside to be catabolized by neuraminidase 3 (Neu3) and thereby compensate for insufficient GM2 breakdown arising from impaired GM2AP-dependent β-HexA activity in this model [51,52]. Future studies will focus on testing efficacy of *scAAV9.hGM2A* on a mouse model (*Gm2a*^−/−^ *Neu3*^−/−^ mice) that exhibits behavioural motor pathologies and lifespan that correspond in severity to patients with infantile ABGM2.

Importantly, we did not observe significant biochemical reduction GM2 accumulation near the end of life (96 weeks post-injection; Figure 4B), though there was a noticeable reduction as compared to controls, which may have been significant if cohort size had been increased. This suggests that the potency of *scAVV9.hGM2A* to reduce GM2 accumulation progressively diminishes after the first assessment period at 14 weeks post-injection. The degree to which potency declines after this time is unclear, and, depending on the rate of this decline, sufficient levels of biochemical correction of GM2 accumulation may be preserved at mid-life or later, which could be clinically significant. Indeed, previous studies suggest that β-HexA activity levels of 10% or greater may be sufficient to achieve a disease-free state [53]. Hence, identification of biochemical correction, even if diminished in potency 14 weeks post-injection, may be relevant given that patients with infantile ABGM2 time points succumb to the disease as early as 4 years of age [21]. The causes of underlying loss of GM2 correction of *scAVV9.hGM2A* by 96 weeks post-injection are unclear. Episomal configurations have been shown to persist for over a decade in non-dividing cell populations [54], although variable stability has been reported [55,56,57]. A more plausible explanation is that there remain a large number of untransduced CNS cells that continue to accumulate GM2, and that this increasing amount of total GM2 accumulation masks the GM2 correction of the population of transduced cells. This explanation is supported by the sub-saturating biodistribution values (i.e., <1 vg copy per cell) in Figure 2 and the appearance of numerous remaining non-corrected cells in Figure 5.

Transgene overexpression has been observed in primate models of TSD, and this was accompanied with neurotoxicity, and failure to correct certain neuropathies [58,59]. Hence, overexpression of GM2AP in the CNS and potentially other organs such as liver and heart could be a clinical concern. This concern is possibly even larger in light of recent studies implicating GM2AP in cancer, diabetes and heart disease [60,61,62,63]. However, we did not notice any significant comorbidities. Tumour incidence, which is the second highest comorbidity observed in this study (the other being weight loss) did not track with dose (11 incidents, 5 in spleen and 3 in liver; Figure 6B); and furthermore, the number of incidents in treated cohorts were comparable with numbers observed in vehicle-treated *Gm2a*^−/−^ and *Gm2a*^+/−^ disease-free cohorts (2 and 1 incident(s), respectively; Figure 6B). Further studies with higher cohort numbers may be needed to clarify whether any comorbidities noted in treated animals are of any significance. Stroke, which is the strongest indictor of heart disease and diabetes tested in this study, was not detected in any of the treated cohorts (Figure 6B). Thus, overexpression of GM2AP in the CNS (seen in the highest dose; 2.0 × 10^11^ vg/mouse), or, potentially, in heart, liver and other visceral organs, is unlikely to be a major safety concern.

The initial study on the development of *Gm2a*^−/−^ mice reported that these mice had normal lifespans [34]. This is consistent with our survival studies, which show that the vehicle-treated *Gm2a^−/−^* and *Gm2a^+/−^* disease-free cohorts exhibit average life expectancies (92 ± 10 vs. 91.0 ± 14 weeks, respectively), similar to that reported for the upper limit of survival of wildtype mice of the same strain (C57BL/6) [64,65]. Our data show that *scAAV9.hGM2A* treatment with all three doses did not significantly impact the average life expectancy of these animals (Figure 6; 87 ± 10 weeks). Consistent with this, *scAAV9.hGM2A* gene therapy appears generally tolerable and non-toxic over the lifespan of treated animals, as exemplified by comorbidity profiles comparable to many of the endpoint pathologies characteristic of aging C57BL/6 mice, which include neoplasms, cysts, and chronic inflammation [66]. A formal toxicology study is warranted to validate our findings.

It is imperative to acknowledge the limitations of this research. Firstly, the *Gm2a*^−/−^ mouse model used in this model displays a relatively mild phenotype, akin to adult-onset ABGM2 [34]. Therefore, we were unable to evaluate the treatment’s impact on behavioural phenotypes or lifespan. Daily oral gavaging, the method of immunosuppressant administration here, could result in oesophageal trauma [67], which could have impacted the overall health of the mice throughout the study. Another noteworthy limitation is the study’s inability to detect GM2 reduction noted at the end of life, as compared to a vehicle-injected *Gm2a*^−/−^ mouse. Technical errors in tissue storage for the histological samples for the vehicle-injected *Gm2a*^−/−^ cohort has hindered the possibility of histologically comparing the treated cohorts to the untreated-disease cohorts at 96 weeks post-injection (Figure 5).

## 4. Materials and Methods

### 4.1. Plasmid Construct

This vector construct was designed as a self-complementary AAV plasmid harbouring a human *GM2A* (*hGM2A*) transgene (Gene ID: 2760) followed by polyadenylation (polyA) sequences and driven by a CBh promoter (Figure 1) [37]. These sequences were flanked by AAV inverted terminal repeat sequences (ITRs); scAAV genomes contain a mutated ITR that is missing the terminal resolution site for continued replication into dsDNA, which enables the production of sc *GM2A* sequences [68,69]. Good-manufacturing-practice-grade AAV viral particles were produced at the University of North Carolina Vector Core (Chapel Hill, NC, USA).

### 4.2. Mice

A murine model of ABGM2 (*Gm2a*^−/−^ mice) was obtained from Jackson Laboratories (003177; B6;129S2-*Gm2a*^tm1Rlp^/J; Bar Harbor, ME, USA). A *Gm2a* null gene was previously engineered in these mice by disrupting a 1 kilobase region consisting of exon 3, intron 3 and a portion of exon 4 with a neomycin resistance cassette [34]. The *Gm2a*^−/−^ mice were interbred with wild type C57BL/6 backgrounds and maintained in the Animal Facility at Queen’s University in Kingston, Ontario. Mice used for experiments were obtained from *Gm2a* ^+/−^ and *Gm2a*^+/−^ or *Gm2a*^−/−^ and *Gm2a*^−/−^ breeding pairs. Progeny genotypes were verified using DNA extracted from ear punch samples obtained at 21 days of age. Briefly, DNA was digested using Q5^®^ High-Fidelity Master Mix (M0491L; New England BioLabs Ltd., Whitby, ON, Canada) and subsequently analysed by PCR using the following primers:

*Gm2a* Mutation Forward 5′-CTTGGGTGGAGAGGCTATTC-3′
*Gm2a* Mutation Reverse 5′-AGGTGAGATGACAGGAGATC-3′
*Gm2a* WT Forward 5′-TACCTACTCACTACCCACGAGC-3′
*Gm2a* WT Reverse 5′-ACACAGAAGAAGAGGCCTGC-3′

Progeny were randomized into experimental genotype cohorts of 6 mice. All experimental procedures were performed in accordance with protocols approved by the Queen’s University Animal Care Committee (Kingston, ON, Canada).

### 4.3. Drug Administration

Two groups of mice were set up in parallel to assess *scAAV9.hGM2A* treatment over a short-term treatment period of 14 weeks and a long-term treatment period of 96 weeks. Each group consisted of 5 cohorts of 6 age-matched mice, giving a total of 30 mice per group. Both groups were intrathecally injected with *scAAV9.hGM2A* or vehicle (i.e., 5% sorbitol, 350 mM NaCl, 2.7 mM KCl and 1.8 mM KH_2_PO_4_) via lumbar puncture at 6 weeks of age. Three cohorts from each group were comprised of *Gm2a*^−/−^ mice; these cohorts received one-time *scAAV9.hGM2A* doses (in 15 μL of vehicle) of either 0.5, 1.0 or 2.0 × 10^11^ vg per mouse. The remaining two cohorts from each group consisted of *Gm2a*^−/−^ homozygote and *Gm2a*^+/−^ heterozygote mice; these cohorts represented untreated affected and disease-free unaffected controls, respectively, and received vehicle. The group of mice treated for the short-term period were euthanized for testing 14 weeks post-injection (20 weeks of age); and the group of mice treated for the long-term period were euthanized for testing 96-weeks post injection (104 week of age or at humane endpoints; see *Euthanizations* below).

### 4.4. Immunosuppressant Administration

Immune responses elicited towards the wild type AAV carrier capsid due to age-dependent seroprevalence of wild type AAV in human populations, and/or to the “non-self” transgene have been observed in clinical trials [70,71,72]. To neutralize similar responses to AAV9 or to the human *GM2A* transgene in *Gm2a*^−/−^ mice, mice were treated with the immunosuppressants rapamycin and prednisone at 5 weeks of age (1 week prior to AAV administration), and thereafter treated daily. Rapamycin and prednisone were administered in combination at varying doses as determined by a previous study [36]. Briefly, rapamycin and prednisone were dissolved in dimethylsulfoxide (Thermo Fisher Scientific, Waltham, MA, USA) and then diluted in 0.9% saline or phosphate buffered saline (PBS), respectively. A loading dose of 300 milligrams (mg) of rapamycin was administered by oral gavage, followed by daily 100 mg treatments for a period of 20 weeks, untapered. Prednisone was administered daily by oral gavage at a dose of 0.24 mg/day for 10 weeks, and then tapered at 0.20 mg/day, 0.16 mg/day, 0.12 mg/day, 0.08 mg/day and 0.04 mg/day for 7 days each.

### 4.5. Behavioural Testing

Open field tests (OFT) and Rotarod (RR) (ActiMot, TSE systems, Berlin, Germany) assessments were carried out on each mouse to assess motor function during short- (20 weeks) and long-term (104 weeks) treatments. Cohorts that were treated with *scAAV9.hGM2A* for 14 weeks were assessed monthly, and cohorts treated for 96 weeks were assessed bimonthly, starting at 8 weeks of age. OFT assesses motor skill by placing animals in a 40 × 40 cm arena and measuring their resting time(s), as well as their movement speed and distance travelled (when not at rest) over a period of 5 min [73]. RR assesses coordination and balance by placing mice on moving cylinders that progressively accelerate from 4 to 40 rotations per minute (RPM) over a period of 5 min. The following parameters indicative of motor function were measured: latency to fall (seconds), end RPM and distance travelled (meters). Each mouse was tested three times on the RR apparatus, with a minimum of 10 min of rest between test trials [73].

### 4.6. Euthanization

Cohorts of mice treated over the short- or long-term treatment periods were euthanized at 20- and 104-weeks age (or when defined humane endpoint criteria were reached), respectively. Humane-endpoint criteria included: (1) loss of 15% of peak mouse weight, or (2) inability of a mouse to right itself. Mice were sacrificed by CO_2_ asphyxiation and blood was collected post-mortem by cardiac puncture. Organs were then perfused with PBS, and immediately thereafter, heart, lungs, liver, spleen, kidney, gonads, muscle, brain and spinal cord were surgically removed for further biochemical and molecular analysis.

### 4.7. Vector Biodistribution

DNA extraction was performed using a DNA extraction kit that was obtained from GeneAid, gSYNC^TM^ DNA Extraction Kit (FroggaBio Inc., Concord, ON, Canada). qPCR was carried out using PowerUp SYBR Green Master Mix (Thermo Fisher, Waltham, MA, USA) on an Applied Biosystems^®^ 7500 Real-Time PCR Systems (Thermo Fisher, Waltham, MA, USA), following the manufacturer’s instructions. To quantify transgene levels, plasmids incorporating the *GM2A* DNA were used as the standard. For mouse genomic DNA quantification, mouse genomic DNA was used as a standard. Primers for the transgene are as follows:

*GM2A* WT forward 5′-TATGGGCTTCCTTGCCACTG-3′
*GM2A* WT reverse 5′-CTCAGGACGCTCTCTATGCG-3′
Mouse LaminB2 primers used for quantification of mouse genomic DNA are as follows:
*LaminB2* WT forward 5′-GGACCCAAGGACTACCTCAAGGG-3′
*LaminB2* WT reverse 5′-AGGGCACCTCCATCTCGGAAAC-3′.
Data is shown as the number of viral genomes (vector DNA copies) per mouse genome (vg/mouse).

### 4.8. Western Blot

Western blot analysis was performed on mid-section brain samples obtained from *Gm2a*^−/−^ and *Gm2a*^+/−^ mice, treated with the *scAAV9.hGM2A*. Protein extracts were subjected to separation by SDS-PAGE and transferred to a nitrocellulose membrane (Bio-Rad Laboratories, Hercules, CA, USA). Tris-buffered saline (TBS) solution containing 5% skim milk was used to block non-specific binding to the membranes. Blocked membranes were probed with antibodies raised against anti-GM2A (Antibody Solutions, Santa, Clara, CA, USA) and with anti-β-actin monoclonal antibodies (Sigma-Aldrich, St. Louis, MI, USA). Membranes were then washed 3 times in TBS containing Tween-20 (TBST) for 10 min each. Immunoblots were then incubated with peroxidase-conjugated secondary antibodies for 1 h at room temperature, washed three times with TBST and then visualized by chemiluminescence (Bio-Rad Laboratories, Hercules, CA, USA). GM2AP expression was quantified using IMAGE J Software (National Institute of Health, Bethesda, MD, USA).

### 4.9. Ganglioside Quantification Assay

GM2 assays were performed to assess the GM2 accumulation within the mid-section of the brain samples of the mice. Gangliosides were extracted from sonicated midbrain samples using methanol and chloroform solvents as described in [74,75]. Next, the samples were diluted in a methanol:chloroform (1:1) solution; 2× the weight of the brains in microliters was added to each pellet of gangliosides. The mixtures were then loaded onto a thin layer chromatography (TLC) plate (Sigma-Aldrich, St. Louis, Missouri, United States) and inserted into a tank containing chloroform, methanol, and calcium chloride (55:45:10). Constituents of the ganglioside mixtures migrate at different speeds on the TLC plate, enabling identification of individual gangliosides. Following development, the plate was sprayed with orcinol (dissolved in 25% sulfuric acid) and heated at 120 °C for 10–15 min until the ganglioside bands were visible (Supplementary Figure S5). Densitometry was used to quantify ganglioside levels using IMAGE J Software (National Institute of Health, Bethesda, MD, United States). A monosialoganglioside mixture (MJS Biolynx Inc., Brockville, ON, Canada) and a *Gm2a* knockout mid-section of the brain samples were used as controls.

### 4.10. Histology

Organ tissues were fixed in 4% paraformaldehyde for 24 h and then transferred to 70% ethanol for a minimum of 24 h. Samples were then shipped to The Center for Phenogenomics (Toronto, ON, Canada) for immunostaining with anti-GM2 antibody (Kyowa Hakko Kirin Co Ltd., New York, NY, USA). Histology sections were quantified based on percent GM2 accumulation in a population of 5–600 cells in the mid-section of the mouse brain. Cells and staining were calculated using QuPath [76].

### 4.11. Statistics

Behavioural statistical analysis was performed using two-way repeated measures ANOVA with the Tukey post hoc test. Ganglioside accumulation and qPCR assays were analysed by a one-way ANOVA with Tukey post hoc test. Kaplan–Meier curves using log rank (Mantel–Cox) tests were used to analyse survival of mice. All statistical analyses were performed in PRISM 9.3.1 (GraphPad, San Diego, CA, USA).

## 5. Conclusions

The goal of this study was to assess the safety and therapeutic potential of *scAAV9.hGM2A* therapy in an ABGM2 mouse model. These data clearly show that *scAAV9.hGM2A* reduces GM2 accumulation when tested at 14 weeks-post-injection, with similar trends at humane end points with all three doses. Taken together, these data provide proof-of-principle that *scAAV9.hGM2A* can stably deliver human *GM2A* transgene to the brain when administered intrathecally, and biochemically reduce GM2 accumulation associated with loss of function of GM2AP in a mouse model of ABGM2. Our data sets a stage for further development of this vector for a human clinical trial of the treatment of ABGM2.

## Data Availability

Not applicable.

## Supplementary Materials

The following supporting information can be downloaded at: https://www.mdpi.com/article/10.3390/ijms24119217/s1.

## References

[1] Mahuran D., Lowden J.A. (1980). The subunit and polypeptide structure of hexosaminidases from human placenta. Can. J. Biochem..

[2] Meier E.M., Schwarzmann G., Fürst W., Sandhoff K. (1991). The human GM2 activator protein. A substrate specific cofactor of beta-hexosaminidase A. J. Biol. Chem..

[3] Maier T., Strater N., Schuette C., Klingenstein R., Sandhoff K., Saenger W. (2003). The X-ray Crystal Structure of Human β-Hexosaminidase B Provides New Insights into Sandhoff Disease. J. Mol. Biol..

[4] Mark B.L., Mahuran D.J., Cherney M.M., Zhao D., Knapp S., James M.N. (2003). Crystal Structure of Human β-Hexosaminidase B: Understanding the Molecular Basis of Sandhoff and Tay–Sachs Disease. J. Mol. Biol..

[5] Lemieux M.J., Mark B.L., Cherney M.M., Withers S.G., Mahuran D.J., James M.N. (2006). Crystallographic Structure of Human β-Hexosaminidase A: Interpretation of Tay-Sachs Mutations and Loss of GM2 Ganglioside Hydrolysis. J. Mol. Biol..

[6] Sharma R., Deng H., Leung A., Mahuran D. (2001). Identification of the 6-Sulfate Binding Site Unique to α-Subunit-Containing Isozymes of Human β-Hexosaminidase. Biochemistry.

[7] Sharma R., Bukovac S., Callahan J., Mahuran D. (2003). A single site in human β-hexosaminidase A binds both 6-sulfate-groups on hexosamines and the sialic acid moiety of GM2 ganglioside. Biochim. Biophys. Acta (BBA)-Mol. Basis Dis..

[8] Zarghooni M., Bukovac S., Tropak M., Callahan J., Mahuran D. (2004). An α-subunit loop structure is required for GM2 activator protein binding by β-hexosaminidase A. Biochem. Biophys. Res. Commun..

[9] McGlynn R., Dobrenis K., Walkley S.U. (2004). Differential subcellular localization of cholesterol, gangliosides, and glycosaminoglycans in murine models of mucopolysaccharide storage disorders. J. Comp. Neurol..

[10] Constantopoulos G., Iqbal K., Dekaban A.S. (1980). Mucopolysaccharidosis Types IH, IS, II and IIIA: Glycosaminoglycans and Lipids of Isolated Brain Cells and Other Fractions from Autopsied Tissues. J. Neurochem..

[11] Constantopoulos G., Dekaban A.S. (1978). Neurochemistry of the Mucopolysaccharidoses: Brain Lipids and Lysosomal Enzymes in Patients with Four Types of Mucopolysaccharidosis and in Normal Controls. J. Neurochem..

[12] Siegel D.A., Walkley S.U. (1994). Growth of Ectopic Dendrites on Cortical Pyramidal Neurons in Neuronal Storage Diseases Correlates with Abnormal Accumulation of GM2 Ganglioside. J. Neurochem..

[13] Zhou S., Davidson C., McGlynn R., Stephney G., Dobrenis K., Vanier M.T., Walkley S.U. (2011). Endosomal/Lysosomal Processing of Gangliosides Affects Neuronal Cholesterol Sequestration in Niemann-Pick Disease Type C. Am. J. Pathol..

[14] Myerowitz R., Hogikyan N.D. (1986). Different Mutations in Ashkenazi Jewish and Non-Jewish French Canadians with Tay-Sachs Disease. Science.

[15] O’Dowd B.F., Klavins M.H., Willard H.F., Gravel R., Lowden J.A., Mahuran D.J. (1986). Molecular heterogeneity in the infantile and juvenile forms of Sandhoff disease (O-variant GM2 gangliosidosis). J. Biol. Chem..

[16] Sandhoff K., Harzer K., Wässle W., Jatzkewitz H. (1971). Enzyme alterations and lipid storage in three variants of tay-sachs disease. J. Neurochem..

[17] Ganne B., Dauriat B., Richard L., Lamari F., Ghorab K., Magy L., Benkirane M., Perani A., Marquet V., Calvas P. (2022). GM2 gangliosidosis AB variant: First case of late onset and review of the literature. Neurol. Sci..

[18] Martins C., Brunel-Guitton C., Lortie A., Gauvin F., Morales C.R., Mitchell G.A., Pshezhetsky A.V. (2017). Atypical juvenile presentation of GM2 gangliosidosis AB in a patient compound-heterozygote for c.259G > T and c.164C > T mutations in the GM2A gene. Mol. Genet. Metab. Rep..

[19] Renaud D., Brodsky M. (2015). GM2-Gangliosidosis, AB Variant: Clinical, Ophthalmological, MRI, and Molecular Findings. JIMD Rep..

[20] Sheth J., Datar C., Mistri M., Bhavsar R., Sheth F., Shah K. (2016). GM2 gangliosidosis AB variant: Novel mutation from India—A case report with a review. BMC Pediatr..

[21] Bley A.E., Giannikopoulos O.A., Hayden D., Kubilus K., Tifft C.J., Eichler F.S. (2011). Natural History of Infantile GM2 Gangliosidosis. Pediatrics.

[22] Maegawa G.H.B., Stockley T., Tropak M., Banwell B., Blaser S., Kok F., Giugliani R., Mahuran D., Clarke J.T. (2006). The Natural History of Juvenile or Subacute GM2 Gangliosidosis: 21 New Cases and Literature Review of 134 Previously Reported. Pediatrics.

[23] Neudorfer O., Pastores G.M., Zeng B.J., Gianutsos J., Zaroff C.M., Kolodny E.H. (2005). Late-onset Tay-Sachs disease: Phenotypic characterization and genotypic correlations in 21 affected patients. Genet. Med..

[24] Toro C., Zainab M., Tifft C.J. (2021). The GM2 gangliosidoses: Unlocking the mysteries of pathogenesis and treatment. Neurosci. Lett..

[25] Choudhury S.R., Hudry E., Maguire C.A., Sena-Esteves M., Breakefield X.O., Grandi P. (2017). Viral vectors for therapy of neurologic diseases. Neuropharmacology.

[26] Leone P., Shera D., McPhee S.W., Francis J.S., Kolodny E.H., Bilaniuk L.T., Wang D.-J., Assadi M., Goldfarb O., Goldman H.W. (2012). Long-Term Follow-Up After Gene Therapy for Canavan Disease. Sci. Transl. Med..

[27] Vandendriessche T., Thorrez L., Acosta-Sanchez A., Petrus I., Wang L., Ma L., De Waele L., Iwasaki Y., Gillijns V., Wilson J.M. (2007). Efficacy and safety of adeno-associated viral vectors based on serotype 8 and 9 vs. lentiviral vectors for hemophilia B gene therapy. J. Thromb. Haemost..

[28] Davidson B.L., Stein C.S., Heth J.A., Martins I., Kotin R.M., Derksen T.A., Zabner J., Ghodsi A., Chiorini J.A. (2000). Recombinant adeno-associated virus type 2, 4, and 5 vectors: Transduction of variant cell types and regions in the mammalian central nervous system. Proc. Natl. Acad. Sci. USA.

[29] McCarty D.M., Young S.M., Samulski R.J. (2004). Integration of Adeno-Associated Virus (AAV) and Recombinant AAV Vectors. Annu. Rev. Genet..

[30] Bey K., Ciron C., Dubreil L., Deniaud J., Ledevin M., Cristini J., Blouin V., Aubourg P., Colle M.-A. (2017). Efficient CNS targeting in adult mice by intrathecal infusion of single-stranded AAV9-GFP for gene therapy of neurological disorders. Gene Ther..

[31] Salegio E.A., Samaranch L., Kells A.P., Mittermeyer G., Sebastian W.S., Zhou S., Beyer J., Forsayeth J., Bankiewicz K.S. (2013). Axonal transport of adeno-associated viral vectors is serotype-dependent. Gene Ther..

[32] Castle M.J., Perlson E., Holzbaur E.L., Wolfe J.H. (2014). Long-distance Axonal Transport of AAV9 Is Driven by Dynein and Kinesin-2 and Is Trafficked in a Highly Motile Rab7-positive Compartment. Mol. Ther..

[33] Green F., Samaranch L., Zhang H.S., Manning-Bog A., Meyer K., Forsayeth J., Bankiewicz K.S. (2016). Axonal transport of AAV9 in nonhuman primate brain. Gene Ther..

[34] Liu Y., Hoffmann A., Grinberg A., Westphal H., McDonald M.P., Miller K.M., Crawley J.N., Sandhoff K., Suzuki K., Proia R.L. (1997). Mouse model of G _M2_ activator deficiency manifests cerebellar pathology and motor impairment. Proc. Natl. Acad. Sci. USA.

[35] Gray S.J., Kalburgi S.N., McCown T.J., Samulski R.J. (2013). Global CNS gene delivery and evasion of anti-AAV-neutralizing antibodies by intrathecal AAV administration in non-human primates. Gene Ther..

[36] Kot S., Karumuthil-Melethil S., Woodley E., Zaric V., Thompson P., Chen Z., Lykken E., Keimel J.G., Kaemmerer W.F., Gray S.J. (2021). Investigating Immune Responses to the scAAV9-*HEXM* Gene Therapy Treatment in Tay–Sachs Disease and Sandhoff Disease Mouse Models. Int. J. Mol. Sci..

[37] Gray S.J., Foti S.B., Schwartz J.W., Bachaboina L., Taylor-Blake B., Coleman J., Ehlers M.D., Zylka M.J., McCown T.J., Samulski R.J. (2011). Optimizing Promoters for Recombinant Adeno-Associated Virus-Mediated Gene Expression in the Peripheral and Central Nervous System Using Self-Complementary Vectors. Hum. Gene Ther..

[38] Réu P., Khosravi A., Bernard S., Mold J.E., Salehpour M., Alkass K., Perl S., Tisdale J., Possnert G., Druid H. (2017). The Lifespan and Turnover of Microglia in the Human Brain. Cell Rep..

[39] Sender R., Milo R. (2021). The distribution of cellular turnover in the human body. Nat. Med..

[40] Demir S.A., Timur Z.K., Ateş N., Martínez L.A., Seyrantepe V. (2020). GM2 ganglioside accumulation causes neuroinflammation and behavioral alterations in a mouse model of early onset Tay-Sachs disease. J. Neuroinflammation.

[41] Gray S.J., Blake B.L., Criswell H.E., Nicolson S.C., Samulski R.J., McCown T.J. (2010). Directed Evolution of a Novel Adeno-associated Virus (AAV) Vector That Crosses the Seizure-compromised Blood–Brain Barrier (BBB). Mol. Ther..

[42] Gray S.J., Matagne V., Bachaboina L., Yadav S., Ojeda S.R., Samulski R.J. (2011). Preclinical Differences of Intravascular AAV9 Delivery to Neurons and Glia: A Comparative Study of Adult Mice and Nonhuman Primates. Mol. Ther..

[43] Woodley E., Osmon K.J., Thompson P., Richmond C., Chen Z., Gray S.J., Walia J.S. (2019). Efficacy of a Bicistronic Vector for Correction of Sandhoff Disease in a Mouse Model. Mol. Ther.-Methods Clin. Dev..

[44] Zincarelli C., Soltys S., Rengo G., Rabinowitz J.E. (2008). Analysis of AAV Serotypes 1–9 Mediated Gene Expression and Tropism in Mice After Systemic Injection. Mol. Ther..

[45] Bradbury A.M., Bagel J.H., Nguyen D., Lykken E.A., Salvador J.P., Jiang X., Swain G.P., Assenmacher C.A., Hendricks I.J., Miyadera K. (2020). Krabbe disease successfully treated via monotherapy of intrathecal gene therapy. J. Clin. Investig..

[46] Bailey R.M., Armao D., Kalburgi S.N., Gray S.J. (2018). Development of Intrathecal AAV9 Gene Therapy for Giant Axonal Neuropathy. Mol. Ther.-Methods Clin. Dev..

[47] Chen X., Dong T., Hu Y., Shaffo F.C., Belur N.R., Mazzulli J.R., Gray S.J. (2022). AAV9/MFSD8 gene therapy is effective in preclinical models of neuronal ceroid lipofuscinosis type 7 disease. J. Clin. Investig..

[48] Hordeaux J., Dubreil L., Robveille C., Deniaud J., Pascal Q., Dequéant B., Pailloux J., Lagalice L., Ledevin M., Babarit C. (2017). Long-term neurologic and cardiac correction by intrathecal gene therapy in Pompe disease. Acta Neuropathol. Commun..

[49] Walia J.S., Osmon K.J., Thompson P., Woodley E., Karumuthil-Melethil S., Heindel C., Keimel J.G., Kaemmerer W.F., Gray S.J. (2022). Treatment of GM2 Gangliosidosis in Adult Sandhoff Mice using an Intravenous Self-Complementary Hexosaminidase Vector. Curr. Gene Ther..

[50] Phaneuf D., Wakamatsu N., Huang J.-Q., Borowski A., Peterson A.C., Fortunato S.R., Ritter G., Igdoura S.A., Morales C.R., Benoit G. (1996). Dramatically Different Phenotypes in Mouse Models of Human Tay-Sachs and Sandhoff Diseases. Hum. Mol. Genet..

[51] Kolter T., Sandhoff K. (1998). Glycosphingolipid degradation and animal models of GM2-gangliosidoses. J. Inherit. Metab. Dis..

[52] Seyrantepe V., Demir S.A., Timur Z.K., Von Gerichten J., Marsching C., Erdemli E., Oztas E., Takahashi K., Yamaguchi K., Ates N. (2018). Murine Sialidase Neu3 facilitates GM2 degradation and bypass in mouse model of Tay-Sachs disease. Exp. Neurol..

[53] Zaccariotto E., Cox T.M. (2018). Genetics and Therapies for GM2 Gangliosidosis. Curr. Gene Ther..

[54] Bulcha J.T., Wang Y., Ma H., Tai P.W.L., Gao G. (2021). Viral vector platforms within the gene therapy landscape. Signal Transduct. Target. Ther..

[55] Nathwani A.C., Tuddenham E.G.D., Rangarajan S., Rosales C., McIntosh J., Linch D.C., Chowdary P., Riddell A., Pie A.J., Harrington C. (2011). Adenovirus-Associated Virus Vector–Mediated Gene Transfer in Hemophilia B. N. Engl. J. Med..

[56] Nathwani A.C., Reiss U.M., Tuddenham E.G., Rosales C., Chowdary P., McIntosh J., Della Peruta M., Lheriteau E., Patel N., Raj D. (2014). Long-Term Safety and Efficacy of Factor IX Gene Therapy in Hemophilia B. N. Engl. J. Med..

[57] Rivera V.M., Gao G.-P., Grant R.L., Schnell M.A., Zoltick P.W., Rozamus L.W., Clackson T., Wilson J.M. (2005). Long-term pharmacologically regulated expression of erythropoietin in primates following AAV-mediated gene transfer. Blood.

[58] Golebiowski D., Van Der Bom I.M.J., Kwon C.-S., Miller A.D., Petrosky K., Bradbury A.M., Maitland S.A., Kühn A.L., Bishop N., Curran E. (2017). Direct Intracranial Injection of AAVrh8 Encoding Monkey β-N-Acetylhexosaminidase Causes Neurotoxicity in the Primate Brain. Hum. Gene Ther..

[59] Leal A.F., Benincore-Flórez E., Solano-Galarza D., Jaramillo R.G.G., Echeverri-Peña O.Y., Suarez D.A., Alméciga-Díaz C.J., Espejo-Mojica A.J. (2020). GM2 Gangliosidoses: Clinical Features, Pathophysiological Aspects, and Current Therapies. Int. J. Mol. Sci..

[60] Ahn H.-S., Kim J.H., Jeong H., Yu J., Yeom J., Song S.H., Kim S.S., Kim I.J., Kim K. (2020). Differential Urinary Proteome Analysis for Predicting Prognosis in Type 2 Diabetes Patients with and without Renal Dysfunction. Int. J. Mol. Sci..

[61] Jiang L., Hao Y., Shao C., Wu Q., Prager B.C., Gimple R.C., Sulli G., Kim L.J., Zhang G., Qiu Z. (2022). ADAR1-mediated RNA editing links ganglioside catabolism to glioblastoma stem cell maintenance. J. Clin. Investig..

[62] Shin J., Kim G., Lee J.W., Lee J.E., Kim Y.S., Yu G.H., Lee S.-T., Ahn S.H., Kim H., Lee C. (2016). Identification of ganglioside GM2 activator playing a role in cancer cell migration through proteomic analysis of breast cancer secretomes. Cancer Sci..

[63] Papaspyridonos M., Smith A., Burnand K.G., Taylor P., Padayachee S., Suckling K.E., James C.H., Greaves D.R., Patel L. (2006). Novel Candidate Genes in Unstable Areas of Human Atherosclerotic Plaques. Arter. Thromb. Vasc. Biol..

[64] Flurkey K., Mcurrer J.M., Harrison D.E., Fox J.G., Davisson M.T., Quimby F.W., Barthold S.W., Newcomer C.E., Smith A.L. (2007). Chapter 20—Mouse Models in Aging Research. The Mouse in Biomedical Research.

[65] Graber T.G., Kim J.-H., Grange R.W., McLoon L.K., Thompson L.V. (2015). C57BL/6 life span study: Age-related declines in muscle power production and contractile velocity. Age.

[66] Pettan-Brewer C., Treuting P.M.M. (2011). Practical pathology of aging mice. Pathobiol. Aging Age-Relat. Dis..

[67] Jones C.P., Boyd K.L., Wallace J.M. (2016). Evaluation of Mice Undergoing Serial Oral Gavage While Awake or Anesthetized. J. Am. Assoc. Lab. Anim. Sci..

[68] McCarty D.M. (2008). Self-complementary AAV Vectors; Advances and Applications. Mol. Ther..

[69] Mccarty D.M., Monahan P.E., Samulski R.J. (2001). Self-complementary recombinant adeno-associated virus (scAAV) vectors promote efficient transduction independently of DNA synthesis. Gene Ther..

[70] Cramer M.L., Shao G., Rodino-Klapac L.R., Chicoine L.G., Martin P.T. (2017). Induction of T-Cell Infiltration and Programmed Death Ligand 2 Expression by Adeno-Associated Virus in Rhesus Macaque Skeletal Muscle and Modulation by Prednisone. Hum. Gene Ther..

[71] Flotte T.R., Cataltepe O., Puri A., Batista A.R., Moser R., McKenna-Yasek D., Douthwright C., Gernoux G., Blackwood M., Mueller C. (2022). AAV gene therapy for Tay-Sachs disease. Nat. Med..

[72] Meliani A., Boisgerault F., Hardet R., Marmier S., Collaud F., Ronzitti G., Leborgne C., Verdera H.C., Sola M.S., Charles S. (2018). Antigen-selective modulation of AAV immunogenicity with tolerogenic rapamycin nanoparticles enables successful vector re-administration. Nat. Commun..

[73] Osmon K.J., Vyas M., Woodley E., Thompson P., Walia J.S. (2018). Battery of Behavioral Tests Assessing General Locomotion, Muscular Strength, and Coordination in Mice. J. Vis. Exp..

[74] Osmon K.J., Woodley E., Thompson P., Ong K., Karumuthil-Melethil S., Keimel J.G., Mark B.L., Mahuran D., Gray S.J., Walia J.S. (2016). Systemic Gene Transfer of a Hexosaminidase Variant Using an scAAV9.47 Vector Corrects G_M2_Gangliosidosis in Sandhoff Mice. Hum. Gene Ther..

[75] Tropak M.B., Bukovac S.W., Rigat B.A., Yonekawa S., Wakarchuk W., Mahuran D.J. (2010). A sensitive fluorescence-based assay for monitoring GM2 ganglioside hydrolysis in live patient cells and their lysates. Glycobiology.

[76] Bankhead P., Loughrey M.B., Fernández J.A., Dombrowski Y., McArt D.G., Dunne P.D., McQuaid S., Gray R.T., Murray L.J., Coleman H.G. (2017). QuPath: Open source software for digital pathology image analysis. Sci. Rep..

