# Supplementary material for: Biochemical Correction of GM2 Ganglioside Accumulation in AB-Variant GM2 Gangliosidosis
Source: Int J Mol Sci. 2023 May 24;24(11):9217. doi: 10.3390/ijms24119217 (PMC10253223; doi:10.3390/ijms24119217)
Supplement: Supplementary file 1 [file ijms-24-09217-s001.zip › ijms-2380612-supplementary.pdf]

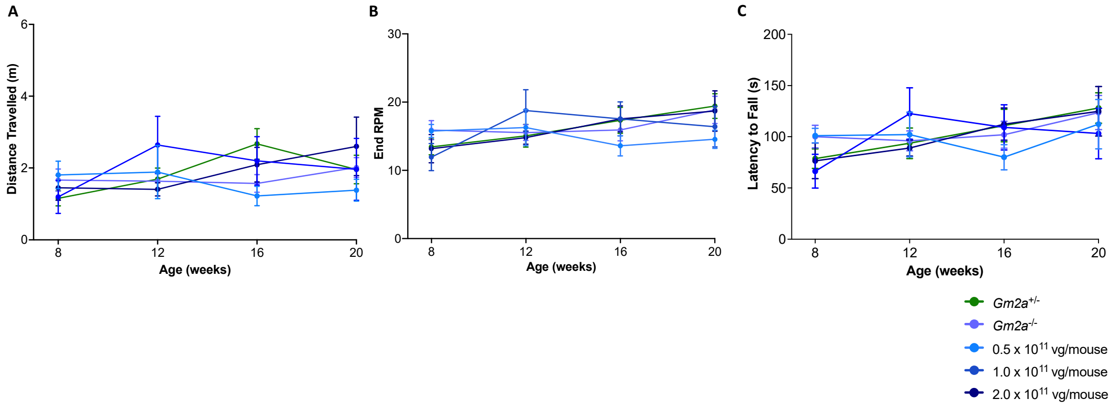

Figure S1. Motor function as assessed by RR tests is unaffected by *scAAV9.hGM2A* treatment between 8-20 weeks of age. To evaluate coordination and balance, testing on a rotating rod (RR) was conducted between 8-20 weeks of age on *scAAV9.GM2A*- or vehicle-treated cohorts (5 cohorts; *n*=6/cohort). The following three parameters were assessed: (a) *distance travelled*, (b) *end* RPM and (b) *latency to fall*. Time points indicate average ◎ SEM (*n* = 6) of the indicated parameter. Significant improvements in motor function (as assessed by tests scores) were not observed between *scAAV9.hGM2A*- or vehicle-treated cohorts (2-way ANOVA) over the period of time tested.

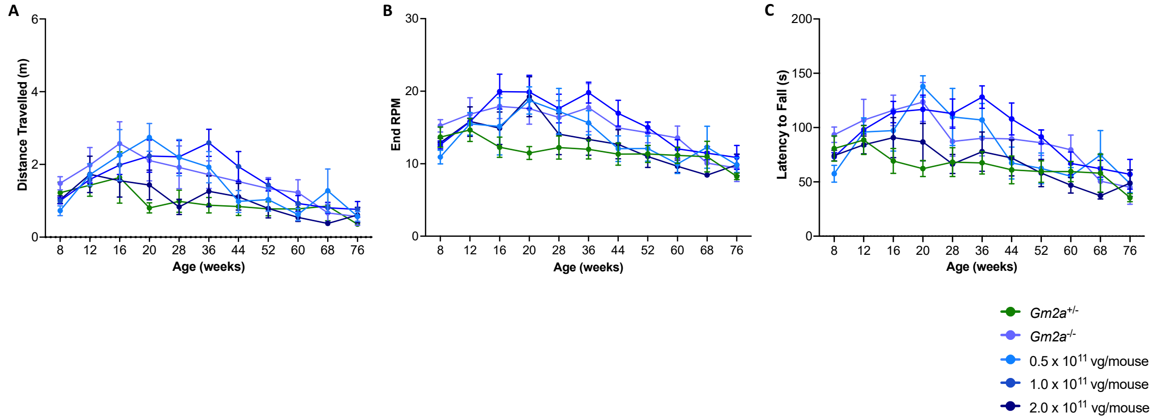

Figure S2. Motor function as assessed by RR tests is unaffected by *scAAV9.hGM2A* treatment between 8-76 weeks of age. To evaluate coordination and balance, testing on a rotating rod (RR) was conducted between 8-76 weeks of age on *scAAV9.hGM2A*- or vehicle-treated cohorts (5 cohorts; *n* = 6/cohort). The following three parameters were assessed: (a) *distance travelled*, (b) *end rotations per minute* (RPM) and (c) *latency to fall*. Significant improvements in parameter scores over the testing period were not observed between *scAAV9.hGM2A*- or vehicle-treated cohorts (2-way ANOVA).

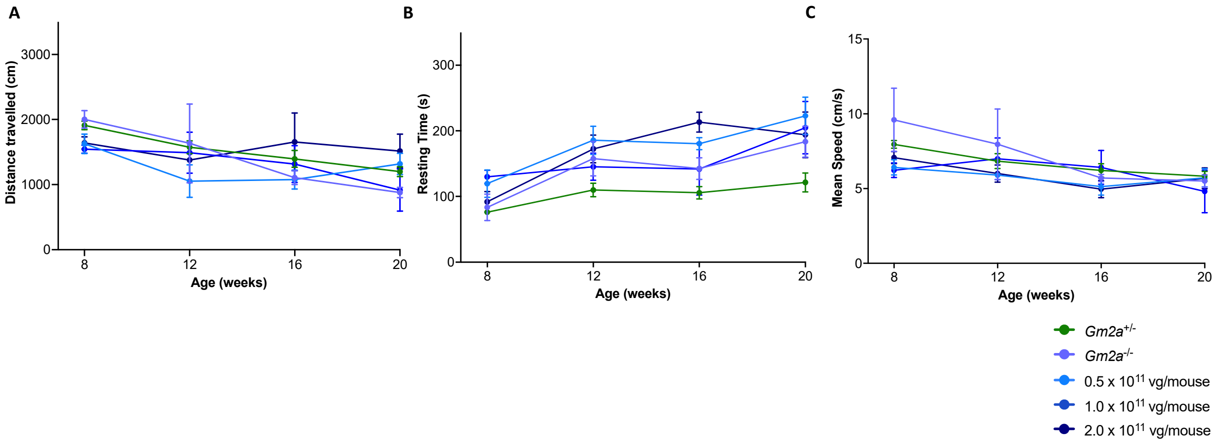

Figure S3. Motor function as assessed by OFT tests is unaffected by *scAAV9.hGM2A* treatment between 8-20 weeks of age. To evaluate coordination and balance, testing in an open field test (OFT) was conducted between 8-20 weeks of age on *scAAV9.hGM2A*- or vehicle-treated cohorts (5 cohorts; *n*=6/cohort). The following three parameters were assessed: (a) *distance travelled*, (b) *resting time* and (c) *mean speed*. Significant improvements in parameter scores over the testing period were not observed between *scAAV9.hGM2A*- or vehicle-treated cohorts (2-way ANOVA).

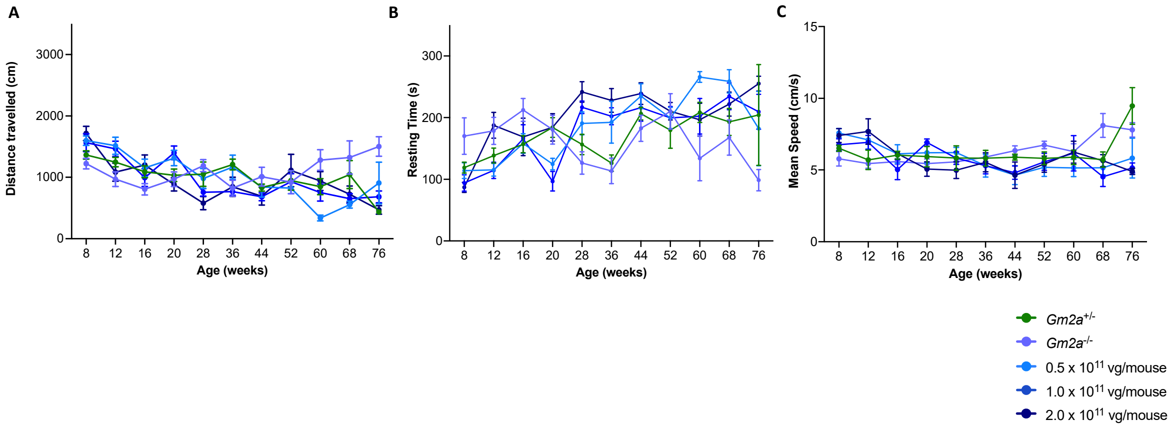

Figure S4. Motor function as assessed by OFT tests is unaffected by *scAAV9.hGM2A* treatment between 8-76 weeks of age. To evaluate coordination and balance, testing in an open field test (OFT) was conducted between 8-76 weeks of age on *scAAV9.hGM2A*- or vehicle-treated cohorts (5 cohorts; *n*=6/cohort). The following three parameters were assessed: (a) *distance travelled*, (b) *resting time* and (c) *mean speed*. Significant improvements in parameter scores over the testing period were not observed between *scAAV9.hGM2A*- or vehicle-treated cohorts (2-way ANOVA).

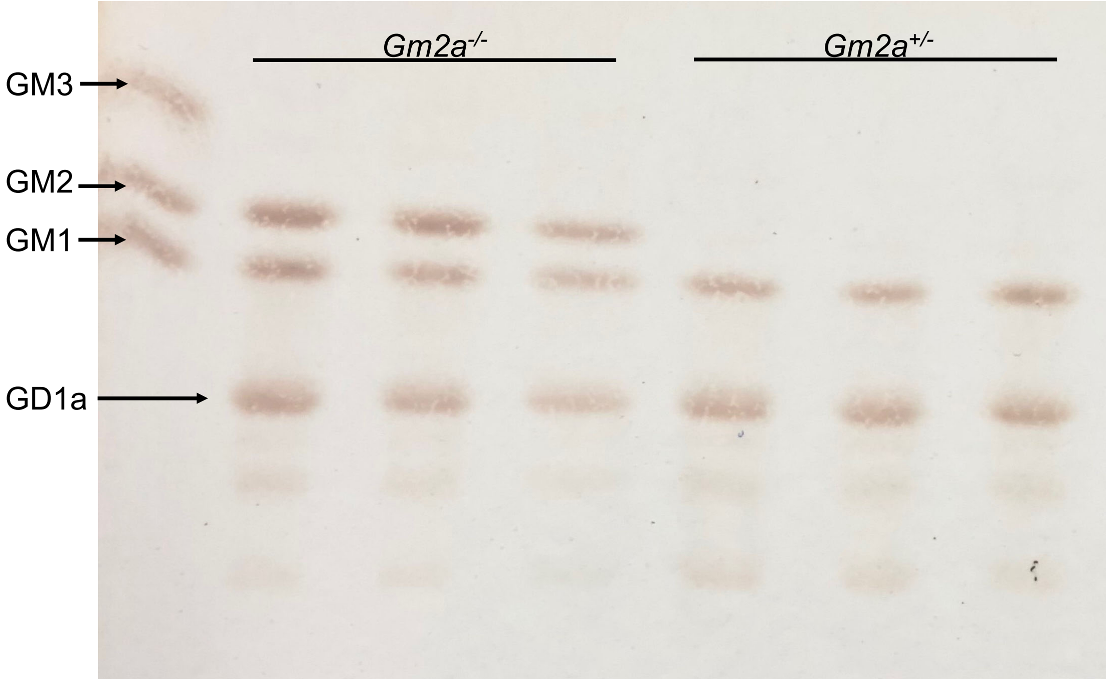

Figure S5. A representative TLC plate for ganglioside quantification (*See Methods 4.9*). This plate includes the monosialoganglioside mixture (lane 1), untreated *Gm2a*$^{-/-}$ mice (lane 2-4), and disease-free *Gm2a*$^{+/-}$ mice (lane 5-7).
